# The Potential for Placental Activation of PPARγ to Improve the Angiogenic Profile in Preeclampsia

**DOI:** 10.3390/cells11213514

**Published:** 2022-11-06

**Authors:** Brooke Grimaldi, Hamid-Reza Kohan-Ghadr, Sascha Drewlo

**Affiliations:** 1 Department of Obstetrics, Gynecology and Reproductive Biology, College of Human Medicine, Michigan State University, Grand Rapids, MI 49503, USA; 2 Biological Sciences Platform, Sunnybrook Health Sciences Centre, Sunnybrook Research Institute, Toronto M4N 3M5, Canada; 3 Department of Obstetrics and Gynecology, Temerty Faculty of Medicine, University of Toronto, Toronto M5G 1E2, Canada

**Keywords:** placenta, preeclampsia, PPARγ, angiogenesis

## Abstract

Preeclampsia (PE) is one of the most common causes of maternal-fetal morbidity and mortality world-wide. While the underlying causes of PE remain elusive, aberrant trophoblast differentiation and function are thought to cause an imbalance of secreted angiogenic proteins resulting in systemic endothelial dysfunction and organ damage in the mother. The placental dysfunction is also characterized by a reduction of the transcription factor, peroxisome proliferator activated receptor γ (PPARγ) which normally promotes trophoblast differentiation and healthy placental function. This study aimed to understand how placental activation of PPARγ effects the secretion of angiogenic proteins and subsequently endothelial function. To study this, healthy and PE placental tissues were cultured with or without the PPARγ agonist, Rosiglitazone, and a Luminex assay was performed to measure secreted proteins from the placenta. To assess the angiogenic effects of placental activation of PPARγ, human umbilical vein endothelial cells (HUVECs) were cultured with the placental conditioned media and the net angiogenic potential of these cells was measured by a tube formation assay. This is the first study to show PPARγ’s beneficial effect on the angiogenic profile in the human preeclamptic placenta through the reduction of anti-angiogenic angiopoietin-2 and soluble endoglin and the upregulation of pro-angiogenic placental growth factor, fibroblast growth factor-2, heparin-binding epidermal growth factor, and follistatin. The changes in the angiogenic profile were supported by the increased angiogenic potential observed in the HUVECs when cultured with conditioned media from rosiglitazone-treated preeclamptic placentas. The restoration of these disrupted pathways by activation of PPARγ in the preeclamptic placenta offers potential to improve placental and endothelial function in PE.

## 1. Introduction

The placenta has a significant role in establishing and maintaining a healthy pregnancy. Abnormal placental development and function is implicated in preeclampsia (PE), which is the leading cause of maternal and fetal morbidity and mortality worldwide [1]. PE clinically manifests around 20 weeks of gestation and is diagnosed based on new onset of maternal hypertension and proteinuria [2]. There is no treatment for PE, and often requires preterm delivery (<37 weeks) as the only treatment option in severe cases. This poses a significant risk to a newborn’s health and is associated with extensive neonatal intensive care costs [3,4]. If untreated, PE can impair maternal hepatic and coagulation systems causing seizures, brain damage, or maternal death. 

Although the etiology of PE remains elusive, evidence suggests insufficient placental perfusion is a major cause of increased inflammation and oxidative stress in the placenta [5]. This environment promotes abnormal villous trophoblast (VT) differentiation, and trophoblast immaturity [6] that further causes abnormal secretion of placental proteins causing an anti-angiogenic state of the placenta [7,8,9,10,11,12,13] and contributing to villous vascular dysfunction [14]. These conditions contribute to systemic endothelial dysfunction which result in the onset of clinical symptoms in the mother. The systemic anti-angiogenic environment in PE additionally poses life-long maternal complications. Nearly half of all women with PE have high blood pressure through 12 weeks post-partum [15,16] and are at risk of developing chronic hypertension within a few years after giving birth [17]. PE poses an even greater risk for cardiovascular disease than smoking [17], and women in the United States who are diagnosed with PE have a 9.4-fold increased risk for cardiovascular-related deaths if the infant is born prior to 34 weeks of gestation [17]. Thus, there is a great need to identify mechanisms of the placental contribution to PE and to establish interventions to dampen maternal sequalae.

Peroxisome proliferator activated receptor-γ (PPARγ) is a widely studied transcription factor, prominently known for its roles in metabolism and adipocyte differentiation [18,19,20,21]. It is also well known that PPARγ acts upstream of several pathways that regulate cell metabolism, anti-inflammatory pathways, and oxidative stress response in the placenta [22]. Our group and others have been uncovering PPARγ’s roles in the placenta, specifically in the regulation of VT differentiation and turnover [22,23,24,25,26,27,28,29]. PPARγ expression and activity is significantly reduced in the PE placenta, and is therefore thought to contribute to PE pathogenesis [25]. Studies have also suggested that activating PPARγ can restore placental function as a potential treatment for PE [23]. Our group has postulated that there could be a connection between the aberrant VT differentiation and the imbalance of secreted proteins from the placenta in PE which cause this anti-angiogenic state. We hypothesize that PPARγ may contribute to maintaining the angiogenic balance in the placenta, and this notion is based on our prior reports showing that activation of PPARγ and its downstream pathways in the human placenta can improve the placental angiogenic environment through downregulating the anti-angiogenic molecule, Soluble fms-like tyrosine kinase 1 [25]. This study further investigates the potential for placental activation of PPARγ to affect the secretion of angiogenic and other growth factor proteins from the placenta that subsequently impact the surrounding endothelium. 

To investigate this, we cultured healthy and PE human placentas in the presence or absence of a PPARγ agonist, Rosiglitazone, and we measured expression of several angiogenic, metabolic, and growth factor proteins (Angiopoeitin-2 (Ang-2), soluble Endoglin (sEng), and Endothelin-1 (ET-1), Placental growth factor (PlGF), Fibroblast growth factor-2 (FGF-2), Epidermal growth factor (EGF), Heparin-binding growth factor (HB-EGF), Follistatin (FST), and Leptin). To better understand if placental activation of PPARγ exerts an effect on the surrounding endothelium, we recapitulated the maternal endothelial response to the placental protein secretion by culturing human umbilical vein endothelial cells (HUVECs) with placental conditioned media. This study emphasizes the benefits of targeting of PPARγ-related pathways as a novel interventional strategy for PE. Although likely other molecules are involved in the process, this first study to report placental activation of PPARγ improves the net placental angiogenic profile and subsequently increases angiogenic potential of the endothelium. Improving maternal endothelial function by managing the negative protein secretion would be beneficial in reducing maternal symptoms in PE.

## 2. Materials and Methods

### 2.1. Tissue Collection

Placentas for this study were provided from two locations at the Research Centre for Women’s and Infants’ Health (RCWIH) BioBank program of Mount Sinai Hospital in Toronto, Canada and from the Women’s Health Center at Spectrum Hospital in Grand Rapids, MI. All placentas collected from Mount Sinai Hospital were done so in accordance with the policies of the Mount Sinai Hospital Research Ethics Board. All placentas collected from Spectrum Health were approved by the IRB waiver of parental consent. Available patient clinical information is listed Table 1. Healthy control placentas (n = 10) were collected from un-complicated pregnancies that delivered by either Cesarean section or vaginal birth between weeks 34–39 of gestation. Placentas collected before 37 weeks were from idiopathic preterm births and the placentas did not contain histological evidence of chorioamnionitis. Pathologic placentas were collected from PE pregnancies (n = 10, Gestation age = 31–37 weeks) or severe PE pregnancies (n = 4; Gestation age = 37–39 weeks) and were delivered either by Cesarean section or vaginal birth. Placentas selected for this study must have met the inclusion criteria for PE/sPE, which is based on the current guidelines for diagnosis of PE, briefly maternal blood pressure >140/90 mm Hg on two occasions longer than 6 h apart, with or without proteinuria and fetal growth restriction [2].

### 2.2. Explant Culture

Placentas were collected using our published methods [25]. In brief, within 2 h post-birth, four 1 cm^3^ cuboidal sections of the placenta were collected and transported to the laboratory in ice-cold HBSS (Hank’s Balanced Salt Solution). The placental sections were immediately processed by rinsing in HBSS and dissected into 20–30 mg sized pieces. The placental tissues were cultures overnight 8% O_2_ with 5% CO_2_ at 37 °C in 500 µL of Dulbecco’s modified Eagle’s medium/Ham’s F-12 nutrient mixture (DMEM/F-12; 1:1; Life Technologies; Grand Island, NY, USA) containing 1% Gibco™ antibiotic-antimycotic. The following day, drug treatments were administered for 18–24 h; 10 µM Rosiglitazone (Selleckchem, Houston, TX, USA) dissolved in dimethyl sulfoxide (DMSO, Sigma Life Sciences, Burlington, MA, USA), DMSO alone was used as a vehicle control. After the culture period for each treatment, the placental conditioned media was collected, snap frozen and stored at −80 °C. Separate conditioned media controls were generated by culturing DMEM/F-12 media overnight at 8% O_2_ with 5% CO_2_ at 37 °C with or without 10 µM Rosiglitazone or DMSO to show the effects of placenta conditioned media. These conditioned media controls are used for controls in the tube formation assay. 

### 2.3. Luminex Assay

The angiogenesis Luminex assay (HAGP1MAG, Millipore Sigma, Burlington, MA, USA) is a multiplex antibody-coated bead based fluorescent assay that allows for quantitative assessment of several proteins using only 25 µL of placental conditioned media. The assay was performed according to manufacturer’s instruction. Briefly, the placental conditioned media was centrifuged at 4500× *g* at 4 °C to pellet any tissue/cell debris and the supernatant was used for the assay. The individual antibody-bead vials were all combined into one solution and pipetted into a 96-well plate and incubated with the conditioned media samples, internal control standard or analyte standards overnight with rocking at 4 °C. The following day, the supernatant contents were removed (analyte:antibody:bead mixtures were contained in the 96-well plate with a plate magnet during removal of supernatant/wash steps). The plate was washed 3 times with wash buffer and then the plate was incubated with the detection antibodies for 1 h at room temperature. Following this, Streptavidin-phycoerythrin was added to each well and incubated for another 30 min at room temperature. The plate was washed 3 times with wash buffer then the sheath fluid was added to each well and the plate was inserted into the Luminex 200 machine. The machine detects the median fluorescent intensity (MFI) of each analyte to measure how much material is present in each sample (pg/mL). The Luminex 200 software performs a 5-point logistic curve analysis of each sample which is compared relative the standard curve. Data from the internal controls were compared to the expected values provided by the kit to ensure the assay was performed properly.

### 2.4. Human Umbilical Vein Endothelial Cell Culture and Tube Formation Assay

Human umbilical vein endothelial cells (HUVECs) were purchased from American Type Culture Collection (ATCC, Manassas, VA, USA) and cultured according to manufacture instruction. Briefly, frozen cells were thawed and seeded in a T-75 flask with F-12K medium (ATCC, Manassas, VA, USA) containing 10% fetal bovine serum (FBS; Life Technologies, Carlsbad, CA, USA), 1% Gibco™ antibiotic-antimycotic, 0.1 mg/mL Heparin and 30 µg/mL Endothelial Cell Growth Supplement. Cell culture medium was changed every 2 days and cells were passaged at 60–70% confluency using 0.25% Trypsin-EDTA solution and 10,000 cells were re-seeded into a new flask. The Tube formation assay was performed based on published protocols [30,31,32,33,34,35,36]. Briefly, the HUVECs were serum-starved for 6 h prior to the tube formation assay and 2 × 96-well plates were coated with 50 µL of Growth-Factor Reduced Matrigel (Corning, Corning, NY, USA) added to each well and incubated at 37 °C for a minimum of 30 min until gel was solidified. HUVECs were removed from the cell culture flasks with trypsin and counted. A 7 mL solution was generated which contained 500,000 cells per mL. 200 µL of this cell suspension was added to separate 1.5 mL Eppendorf tubes, each tube corresponded to a different treatment, listed in Table 2. 800 µL of the control medium or placental conditioned medium (spun at 4500× *g* at 4 °C for 10 min) was added to the 1.5 mL Eppendorf tubes that each contained 100,000 HUVECs. 100 µL of the HUVEC-conditioned media solution was added per well in the 96-well plate. There was a minimum of 7 wells (technical replicates) for each treatment. The plates were incubated for 18 h in 20% O_2_ with 5% CO_2_ at 37 °C then phase contrast images were captured on an inverted microscope using the 4× objective. Images were imported into the ImageJ software [37] and the images were analyzed using the Angiogenesis Analyzer macro plugin [30]. To quantify angiogenesis, the images were segmented and skeletonized, and the trees were analyzed to provide quantitative assessment of the number of nodes, junctions, meshes and total branching length present in the HUVECs.

### 2.5. Statistical Analysis 

All statistical analysis was performed with GraphPad Prism 7.0 software. Raw expressions were analyzed by student’s *t*-test, after determination if samples are normally distributed and an F-test was applied to determine variances between groups which was then used in the parameters for the *t*-test. *p* < 0.05 is considered significant and is indicated with (*) on each graph. Data is reported as Mean ± S.E.M. All sample numbers are reported as per group, for example, n = 6 designates 6 samples per treatment/group. 

## 3. Results

### 3.1. Rosiglitazone Has a Significant Impact on Angiogenic and Growth Factor Protein Secretion from the Preeclamptic Placenta

**Figure 1:**
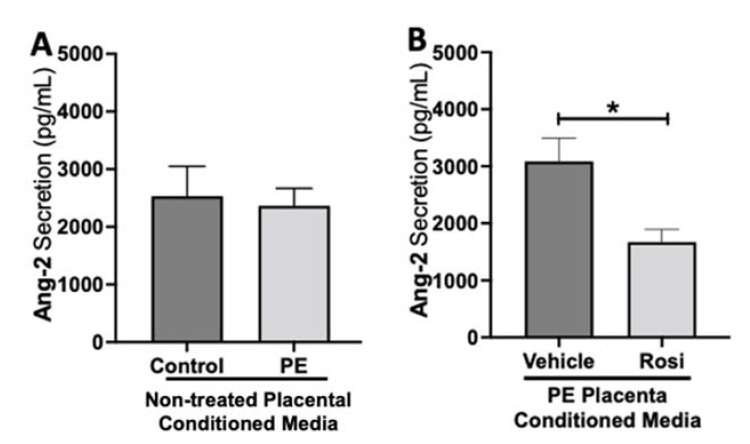
Angiopoeitin-2 secretion is reduced in Rosiglitazone-treated preeclamptic placentas. Angiopoeitin-2 (Ang-2) levels were measured via Luminex assay from conditioned media from non-treated control and preeclamptic (PE) placentas (**A**) and vehicle- or Rosiglitazone (Rosi)-treated PE placentas (**B**). There was no significant difference in Ang-2 secretion between PE and control placentas (**A**, n > 10). Rosi-treated PE placentas show a significant reduction of Ang-2 secretion in comparison to the vehicle control (**B**, n = 14). (Protein secretion was measured by a Luminex assay where experimental values were determined relative to a standard curve. Statistical analysis was performed by student’s *t*-test to determine significant differences between groups, * *p* < 0.05, bar plots and data reported are reported as mean pg/mL values ± SEM).

To test if placental activation of PPARγ influences angiogenic protein secretion, preeclamptic placentas treated with or without Rosiglitazone and vehicle, as well as non-treated healthy placentas were cultured for 24 h, and the conditioned medium was used for Luminex assay. Our luminex data shows no significant differences in Angiopoietin-2 (Ang-2) secretion levels between the non-treated control and non-treated PE placentas (2532 ± 517.6 pg/mL vs. 2365 ± 303.7 pg/mL, n > 10, *p* > 0.05, Figure 1A). However, we observed a significant reduction in Ang-2 secretion in Rosiglitazone-treated PE placentas when compared to the vehicle (1671 ± 223 pg/mL vs. 3086 ± 407 pg/mL, n = 14, *p* = 0.04, Figure 1B). 

**Figure 2:**
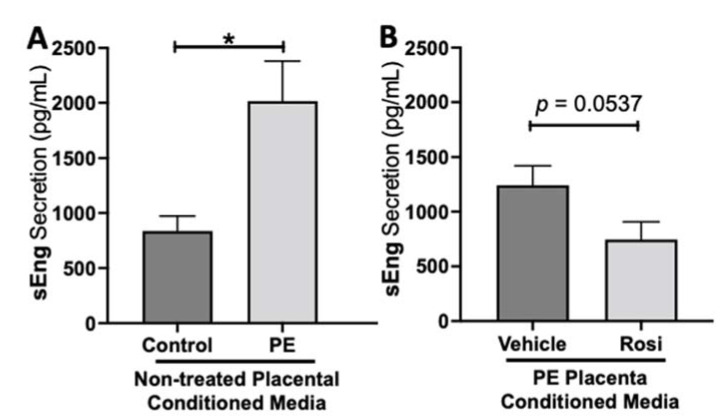
Soluble Endoglin secretion is increased in the preeclamptic placenta but reduced after Rosiglitazone treatment. Soluble Endoglin (sEng) levels were measured via Luminex assay from conditioned media from non-treated control and preeclamptic (PE) placentas (**A**) and vehicle- or Rosiglitazone (Rosi)-treated PE placentas (**B**). There was a significant upregulation of sEng secretion in PE compared to control placentas (**A**, n > 10). Rosi-treated PE placentas show a reduction of sEng secretion however this was not statistically significant when compared to the vehicle control (**B**, n = 14). (Protein secretion was measured by a Luminex assay where experimental values were determined relative to a standard curve. Statistical analysis was performed by student’s *t*-test to determine significant differences between groups, * *p* < 0.05, bar plots and data reported are reported as mean pg/mL values ± SEM).

We observed a significant upregulation of soluble Endoglin (sEng) from the preeclamptic placentas in comparison to the healthy control placentas (2017 ± 364 pg/mL vs. 836 ± 135 pg/mL, n > 10, *p* = 0.0164, Figure 2A). We also observed a reduction of sEng in the Rosiglitazone-treated PE placentas in comparison to the vehicle control however it was not statistically significant (745 ± 161.5 pg/mL vs. 1242 ± 179 pg/mL, n = 14, *p* = 0.0537, Figure 2B). 

**Figure 3:**
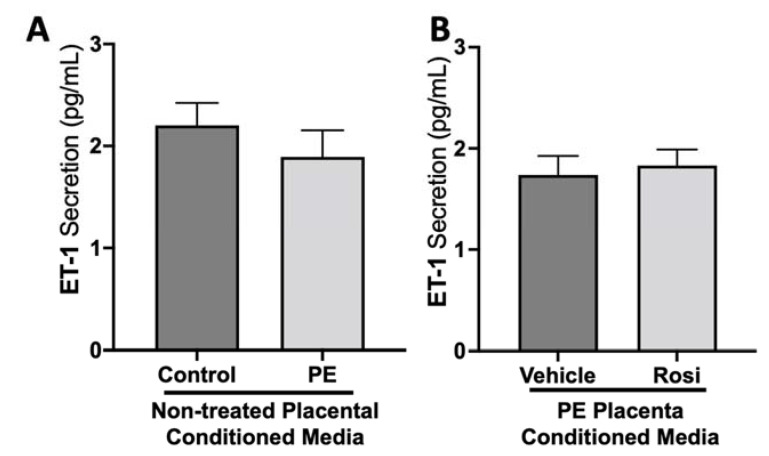
There are no significant differences in Endothelin-1 secretion from healthy or preeclamptic placentas with or without drug treatment. Secretion of Endothelin-1 was measured via Luminex assay from conditioned media from non-treated control and preeclamptic (PE) placentas (**A**) and vehicle- or Rosiglitazone (Rosi)-treated PE placentas (**B**). There was no significant change in ET-1 secretion between PE and control placentas (**A**, n > 10) and between vehicle and Rosi-treated PE placentas in the PE compared to control placentas however this was not statistically significant (**B**, n = 14). (Protein secretion was measured by a Luminex assay where experimental values were determined relative to a standard curve. Statistical analysis was performed by student’s *t*-test to determine significant differences between groups, bar plots and data reported are reported as mean pg/mL values ± SEM).

We did not observe a significant difference in Endothelin-1 (ET-1) secretion from control compared to preeclamptic placentas (2.2 ± 0.2 pg/mL vs. 1.9 ± 0.25 pg/mL, n > 10, *p* > 0.05, Figure 3A). Further, there was no change in ET-1 secretion from Rosiglitazone-treated PE placentas compared to the vehicle control (1.83 ± 0.15 pg/mL vs. 1.74 ± 0.19 pg/mL, n = 14, *p* > 0.05, Figure 3B). 

**Figure 4:**
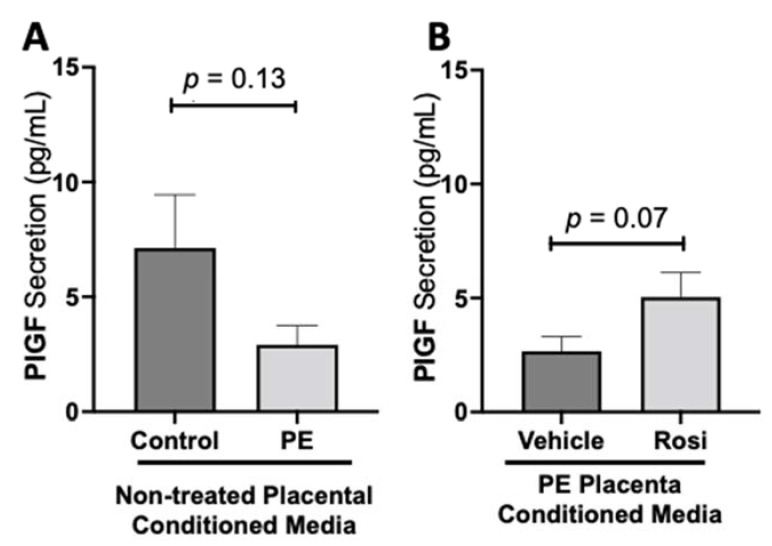
There is a decreasing trend in placental growth factor secretion in the preeclamptic placenta that is partially rescued by Rosiglitazone treatment. Secretion of Placental growth factor (PlGF) were measured via Luminex assay from conditioned media from non-treated control and preeclamptic (PE) placentas (**A**) and vehicle- or Rosiglitazone (Rosi)-treated PE placentas (**B**). There was a trending decrease of PlGF secretion in the PE compared to control placentas however this was not statistically significant (**A**, n > 10). Rosi-treated PE placentas show an increase of PlGF secretion however this was not statistically significant when compared to the vehicle control (**B**, n = 14). (Protein secretion was measured by a Luminex assay where experimental values were determined relative to a standard curve. Statistical analysis was performed by student’s *t*-test to determine significant differences between groups, bar plots and data reported are reported as mean pg/mL values ± SEM).

We observed a decreasing trend of placental growth factor (PlGF) secretion from PE placentas however, this was not statistically significant in comparison to the control placentas (2.913 ± 0.82 pg/mL vs. 7.12 ± 2.3 pg/mL, n > 10, *p* = 0.13, Figure 4A). We observed an increase of PlGF secretion in the Rosiglitazone-treated PE placentas however, it was not statistically significant in comparison to the vehicle (4.765 ± 1 pg/mL vs. 2.668 ± 0.6 pg/mL, n = 14, *p* = 0.07, Figure 4B). 

**Figure 5:**
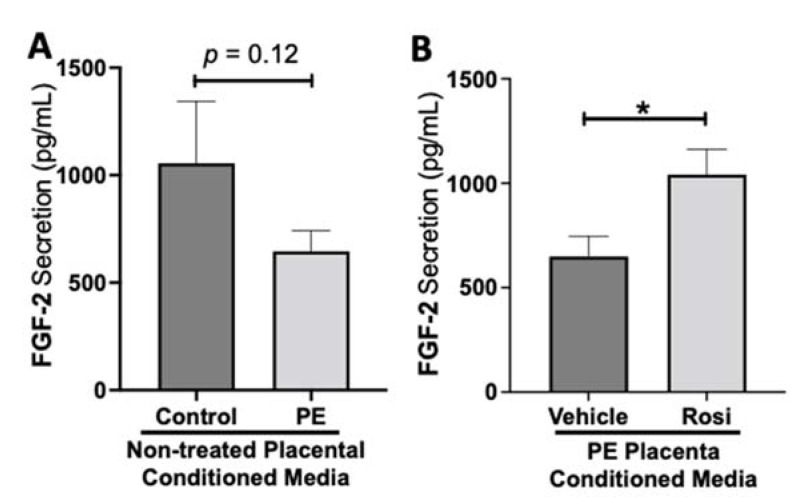
Fibroblast Growth Factor 2 shows reduced secretion from the preeclamptic placenta that is reversed by Rosiglitazone treatment. Secretion of Fibroblast Growth Factor 2 (FGF-2) was measured via Luminex assay from conditioned media from non-treated control and preeclamptic (PE) placentas (**A**) and vehicle- or Rosiglitazone (Rosi)-treated PE placentas (**B**). FGF-2 secretion appears to be reduced in preeclamptic placenta however this decrease is not statistically different from control placentas (**A**, n > 10). Rosi-treated PE placentas show a significant increase in FGF-2 secretion compared to the vehicle control (**B**, n = 14). (Protein secretion was measured by a Luminex assay where experimental values were determined relative to a standard curve. Statistical analysis was performed by student’s *t*-test to determine significant differences between groups, * *p* < 0.05, bar plots and data reported are reported as mean pg/mL values ± SEM).

While we observed a decreasing trend of fibroblast growth factor (FGF-2) secretion from preeclamptic placentas, this was not statistically significant change in FGF-2 secretion in preeclamptic compared to healthy control placentas (977 ± 266 pg/mL vs. 646 ± 96 pg/mL, n > 10, *p* = 0.12, Figure 5A). However, Rosiglitazone caused a significant increase of FGF-2 secretion in PE placentas compared to vehicle treatment (1041 ± 121 pg/mL vs. 649 ± 97 pg/mL, n = 14, *p* = 0.04, Figure 5B). 

**Figure 6:**
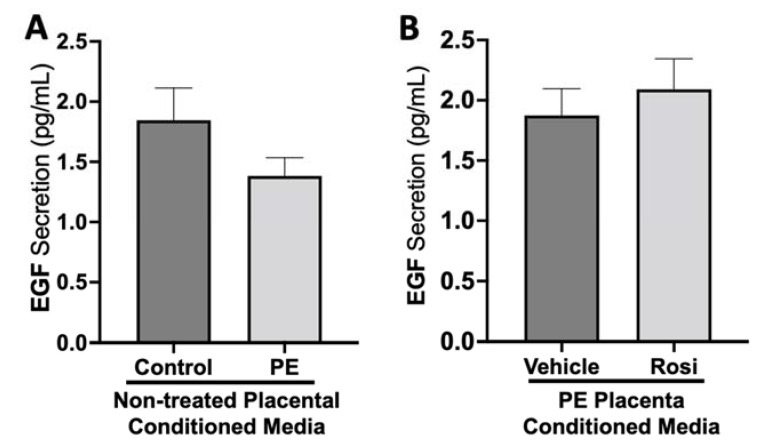
There are no significant changes in Epidermal Growth Factor secretion between healthy and preeclamptic placentas treated with or without Rosiglitazone. Secretion of Epidermal Growth Factor (EGF) was measured via Luminex assay from conditioned media from non-treated control and preeclamptic (PE) placentas (**A**) and vehicle- or Rosiglitazone (Rosi)-treated PE placentas (**B**). There was no significant change in EGF secretion between PE and control placentas (**A**, n > 10) and between vehicle and Rosi-treated PE placentas in the PE compared to control placentas however this was not statistically significant (**B**, n = 14). (Protein secretion was measured by a Luminex assay where experimental values were determined relative to a standard curve. Statistical analysis was performed by student’s t-test to determine significant differences between groups, bar plots and data reported are reported as mean pg/mL values ± SEM).

There was no significant change in epidermal growth factor (EGF) secretion between preeclamptic and control placentas (1.38 ± 0.15 pg/mL vs. 1.84 ± 0.27 pg/mL, n > 10, *p* > 0.05, Figure 6A). There were also no measurable changes of EGF secretion from Rosiglitazone-treated PE placentas in comparison to vehicle-treated PE placentas (2.1 ± 0.25 pg/mL vs. 1.8 ± 0.22 pg/mL, n = 14, *p* > 0.05, Figure 6B). 

**Figure 7:**
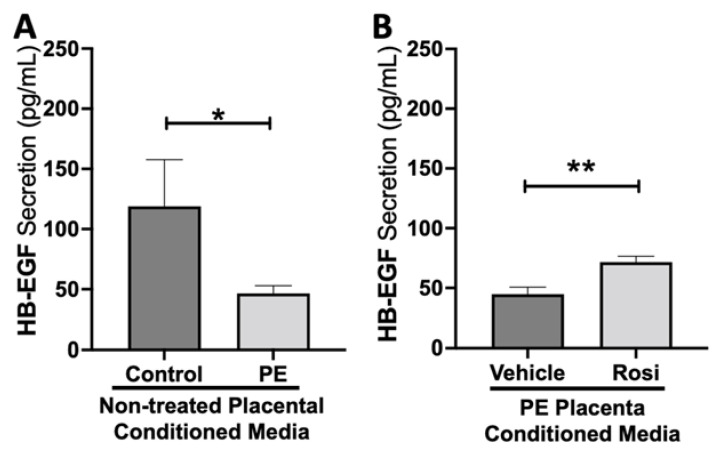
Heparin-Binding Epidermal Growth Factor shows reduced secretion from the preeclamptic placenta but is reversed by Rosiglitazone treatment. Secretion of Heparin-Binding Epidermal Growth Factor (HB-EGF) was measured via Luminex assay from conditioned media from non-treated control and preeclamptic (PE) placentas (**A**) and vehicle- or Rosiglitazone (Rosi)-treated PE placentas (**B**). There was a significant reduction of HB-EGF secretion from the PE placenta compared to control (**A**, n > 6). Rosi-treated PE placentas show a significant increase in HB-EGF secretion compared to the vehicle control (**B**, n = 10). (Protein secretion was measured by a Luminex assay where experimental values were determined relative to a standard curve. Statistical analysis was performed by student’s *t*-test to determine significant differences between groups, * *p* < 0.05, ** *p* < 0.01, bar plots and data reported are reported as mean pg/mL values ± SEM).

Our data indicates that secretion of heparin-binding epidermal growth factor (HB-EGF) is significantly reduced in the PE placenta in comparison to controls (46.5 ± 6.4 pg/mL vs. 119 ± 38 pg/mL, n > 10, *p* = 0.05, Figure 7A). However, HB-EGF secretion was restored when treated with Rosiglitazone in comparison to the vehicle control (71.6 ± 4.9 pg/mL vs. 44.8 ± 6 pg/mL, n = 14, *p* = 0.0027, Figure 7B). 

**Figure 8:**
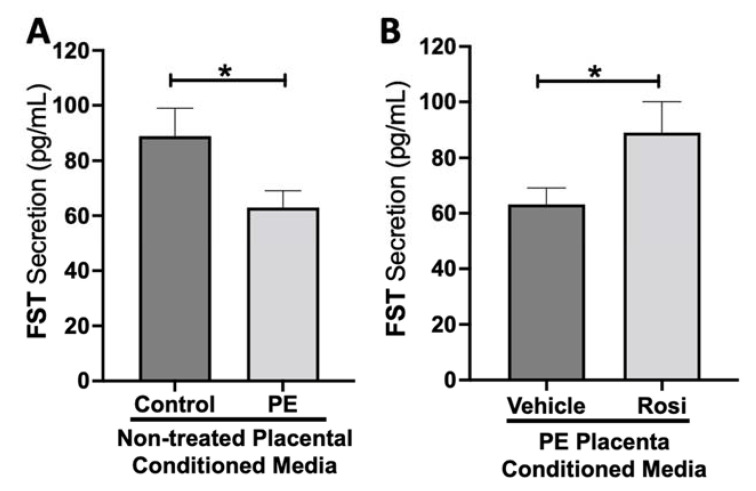
Follistatin shows reduced secretion from the preeclamptic placenta but is reversed by Rosiglitazone. Secretion of Follistatin (FST) was measured via Luminex assay from conditioned media from non-treated control and preeclamptic (PE) placentas (**A**) and vehicle- or Rosiglitazone (Rosi)-treated PE placentas (**B**). FST secretion was significantly reduced in the PE placenta compared to control placentas (**A**, n > 10) however, Rosi treatment led to a significantly increased secretion of FST from the PE placenta compared to vehicle-treated PE placentas (**B**, n = 14). (Protein secretion was measured by a Luminex assay where experimental values were determined relative to a standard curve. Statistical analysis was performed by student’s *t*-test to determine significant differences between groups, * *p* < 0.05, bar plots and data reported are reported as mean pg/mL values ± SEM).

Our data shows a significant reduction of Follistatin (FST) secretion from the PE placenta in comparison to control (89 ± 9 pg/mL vs. 63 ± 6 pg/mL, n > 10, *p* = 0.034, Figure 8A). Rosiglitazone caused a significant increase in FST secretion from the PE placenta in comparison to the vehicle control (89 ± 10 pg/mL vs. 63 ± 5 pg/mL, n = 14, *p* = 0.044, Figure 8B). 

**Figure 9:**
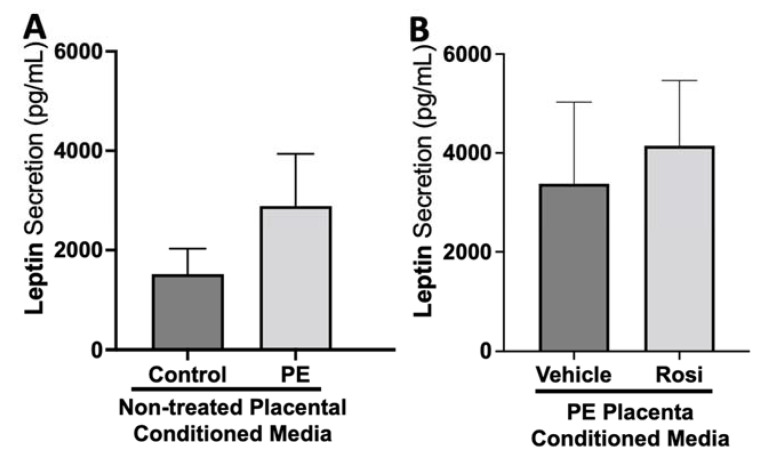
There are no significant changes in Leptin secretion between healthy and preeclamptic placentas with or without drug treatment. Secretion of Leptin was measured via Luminex assay from conditioned media from non-treated control and preeclamptic (PE) placentas (**A**) and vehicle- or Rosiglitazone (Rosi)-treated PE placentas (**B**). Although there was an increasing trend of Leptin secretion from PE placentas, this was not statistically different from the control placentas (**A**, n > 10). There were no significant changes in Leptin secretion between vehicle and Rosi-treated PE placentas (**B**, n = 14). (Protein secretion was measured by a Luminex assay where experimental values were determined relative to a standard curve. Statistical analysis was performed by student’s *t*-test to determine significant differences between groups, bar plots and data reported are reported as mean pg/mL values ± SEM).

There appeared to be a greater secretion of Leptin from the PE placenta in comparison to the control placentas although this was not statistically significant (2889 ± 1047 pg/mL vs. 1517 ± 514 pg/mL, n > 10, *p* > 0.05, Figure 9A). Rosiglitazone-treated PE placentas did not show a significant change in Leptin secretion in comparison to the vehicle-treated PE placentas (4146 ± 1320 pg/mL vs. 3376 ± 1651 pg/mL, n = 14, *p* > 0.05, Figure 9B). 

### 3.2. Tube Formation Assays Reveals a Pro-Angiogenic Effect from Rosiglitazone-Treated Preeclamptic Placentas

The endothelial tube formation is an assessment of angiogenesis through the measurement of nodes, junctions, meshes, and total branching length of the HUVECs when cultured with placental conditioned media or control media. The nodes from the HUVEC structure represent the location of two endothelial branches. Junctions are determined when a node has three or more branches that are intersecting. The total branching length is quantified as the sum of all the branch lengths per image. The ‘mesh’ is used to describe the general HUVEC structure (typically appearing in a spider-web like structure) and is calculated by measuring the areas enclosed by the branches. In total, these measurements can relate to the potential for the HUVECs to undergo tube formation, which we refer to as ‘angiogenic potential’. 

**Figure 10:**
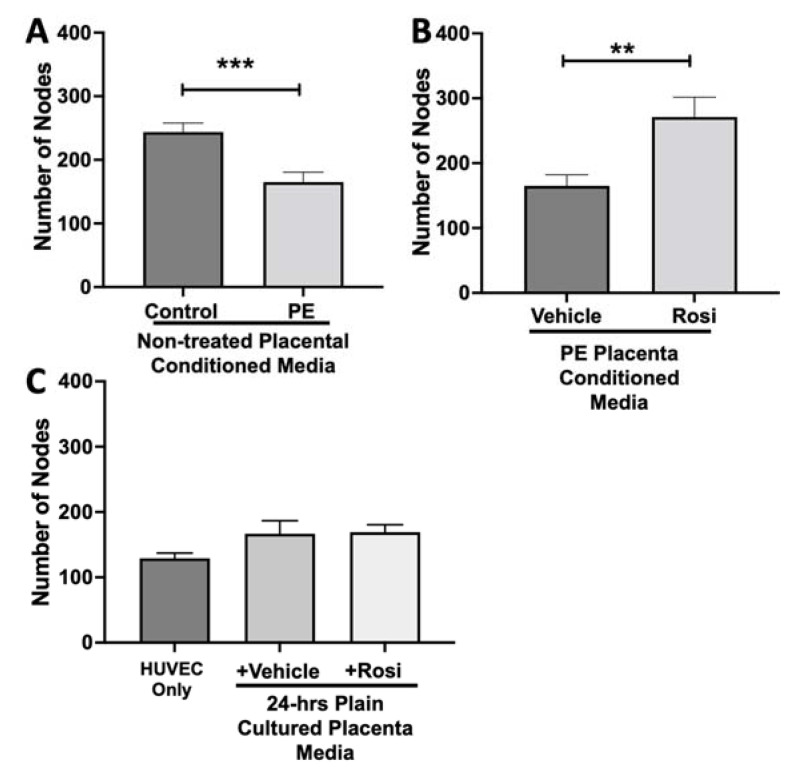
There is a significant reduction in the number of nodes present in the HUVECs cultured with preeclamptic conditioned media, but this is reversed in Rosiglitazone-treated placentas. HUVECs were cultured with conditioned media on matrigel, and the number of nodes present was calculated by the Image J Angiogenesis Analyzer tool (30). HUVECs cultured with conditioned media from preeclamptic (PE) placentas show significantly reduced number of nodes as compared to healthy control placentas (**A**, n =6). Conditioned media from Rosiglitazone (Rosi)-treated PE placentas led to a significant increase in the number of nodes present in the HUVECs compared to the conditioned media from the vehicle-treated PE placentas (**B**, n = 6). Rosi and vehicle were cultured in placental media without any tissues for 24 h then applied to the HUVECs. Additionally, HUVECs cultured with standard full-serum media (HUVEC Only) all served as controls for this experiment. There were no significant changes in the number of nodes present among the HUVEC Only, Rosi, and vehicle conditioned media controls (**C**, n = 6). (Statistical analysis was performed by student’s *t*-test to determine significant differences between groups, ** *p* < 0.01, *** *p* < 0.001, bar plots and data reported are reported as numerical values ± SEM).

We observed a significant reduction in the number of nodes present in the HUVECs treated with PE conditioned media compared to control placenta conditioned media (165 ± 15 vs. 243 ± 14, n = 6, *p* = 0.0004, Figure 10A). However, there was a statistically significant increase in the number of nodes present in the HUVECs cultured with Rosiglitazone-treated placental conditioned media in comparison to the conditioned media from vehicle-treated placentas (271 ± 30 vs. 165 ± 17, n = 6, *p* = 0.0032, Figure 10B). We observed no significant changes in the number of nodes produced from the HUVEC positive control when compared to the HUVECs cultured with the vehicle and Rosiglitazone controls (139 ± 8 vs. 167 ± 20 vs. 16 9± 12, n = 6, *p* > 0.05, Figure 10C). 

**Figure 11:**
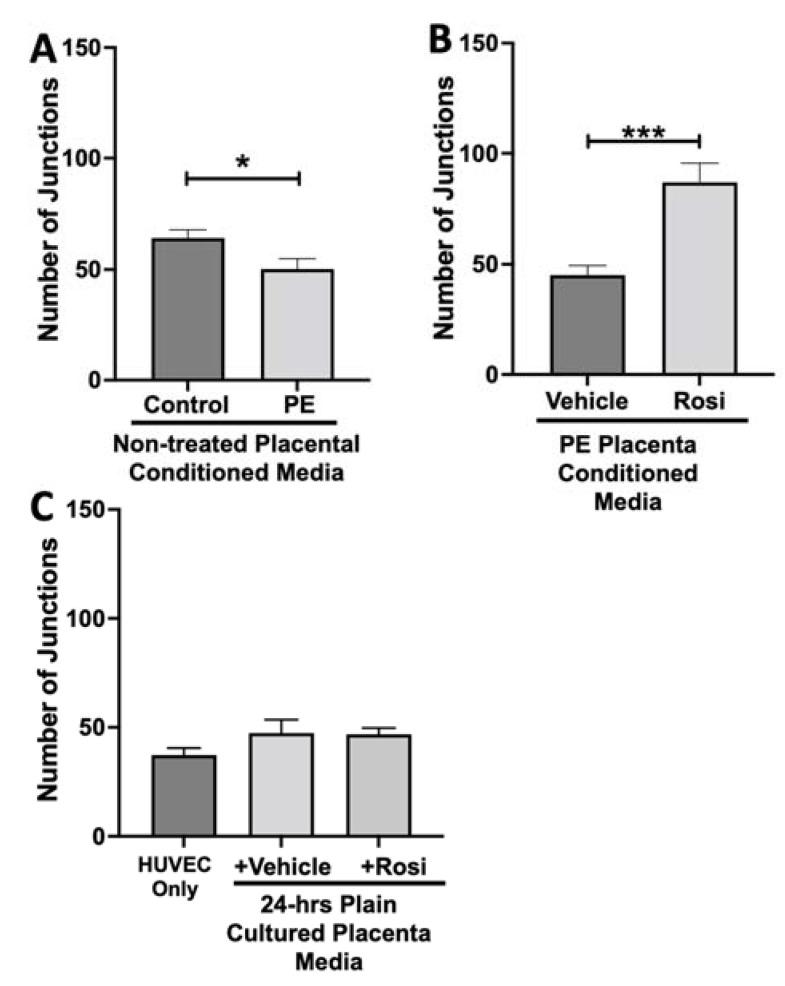
There is a reduction of junctions present in HUVECs cultured with preeclamptic placental conditioned media and this was significantly increased after culture with Rosiglitazone-treated preeclamptic placentas. HUVECs were cultured with conditioned media on matrigel, and the number of junctions present was calculated by the Image J Angiogenesis Analyzer tool (30). Conditioned media from preeclamptic (PE) placentas show significantly reduced number of junctions as compared to healthy control placentas (**A**, n = 6). Conditioned media from Rosiglitazone (Rosi)-treated PE placentas led to a significant increase in the number of junctions present in the HUVECs compared to the conditioned media from the vehicle-treated PE placentas (**B**, n = 6). Rosi and vehicle were cultured in placental media without any tissues for 24 h then applied to the HUVECs. Additionally, HUVECs cultured with standard full-serum media (HUVEC Only) all as controls for this experiment. There were no significant changes in the number of junctions present among the HUVEC Only, Rosi, and vehicle conditioned media controls (**C**, n = 6). (Statistical analysis was performed by student’s *t*-test to determine significant differences between groups, * *p* < 0.05, *** *p* < 0.001, bar plots and data reported are reported as numerical values ± SEM).

The number of junctions among the HUVECs was also measured and is shown to be significantly reduced when cultured with conditioned media from non-treated PE compared to control placentas (50 ± 4 vs. 64 ± 3, n = 6, *p* = 0.02, Figure 11A). There was a significant increase in the number of junctions in the HUVECs after culture with conditioned media from Rosiglitazone-treated PE placentas as compared to vehicle-treated PE placentas (87 ± 8 vs. 45 ± 4, n = 6, *p* < 0.0001, Figure 11B). There were no significant differences in the number of junctions shown in the HUVEC Only control as compared to the vehicle and Rosiglitazone conditioned media controls (37 ± 3 vs. 47 ± 6 vs. 46 ± 3, n = 6, *p* > 0.05, Figure 11C). 

**Figure 12:**
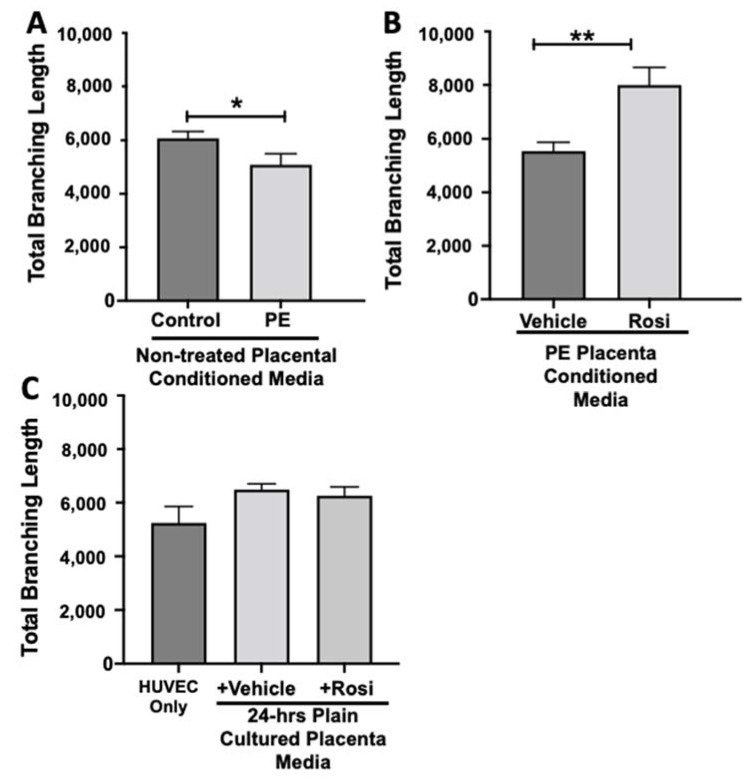
There is a reduction of the total branching length present in HUVECs cultured with preeclamptic placental conditioned media, but this was significantly increased after culture with Rosiglitazone-treated preeclamptic placentas. HUVECs were cultured with conditioned media on matrigel, and the total branching length was calculated by the Image J Angiogenesis Analyzer tool (30). Conditioned media from preeclamptic (PE) placentas show significantly reduced total branching length as compared to healthy control placentas (**A**, n = 6). Conditioned media from Rosiglitazone (Rosi)-treated PE placentas led to a significant increase in the total branching length present in the HUVECs compared to the conditioned media from the vehicle-treated PE placentas (**B**, n = 6). Rosiglitazone and vehicle were cultured in placental media without any tissues for 24 h along with HUVECs cultured with standard full-serum media (HUVEC Only) all served as controls for this experiment. There were not any significant changes in the total branching length among the HUVEC Only control, Rosi, and vehicle conditioned media controls (**C**, n = 6). (Statistical analysis was performed by student’s *t*-test to determine significant differences between groups, * *p* < 0.05, ** *p* < 0.01, bar plots and data reported are reported as numerical values ± SEM).

Our data shows that HUVECs cultured with conditioned media from PE placentas show significantly reduced total branching length in comparison to the conditioned media from the control placentas (5085 ± 414 vs. 6074 ± 257 relative pixel values, n = 6, *p* = 0.03, Figure 12A). However, the conditioned medium from the Rosiglitazone-treated PE placentas led to a significant increase in the total branching length as compared to the conditioned media from the vehicle-treated PE placentas (7994 ± 662 vs. 5538 ± 337 relative pixel values, n = 6, *p* = 0.0013, Figure 12B). There was no significant difference in total branching length between the HUVEC Only control, the vehicle conditioned media, and Rosiglitazone conditioned media controls (5238 ± 621, 6401 ± 554, 5885 ± 336 relative pixel values, n = 6, *p* > 0.05, Figure 12C). 

**Figure 13:**
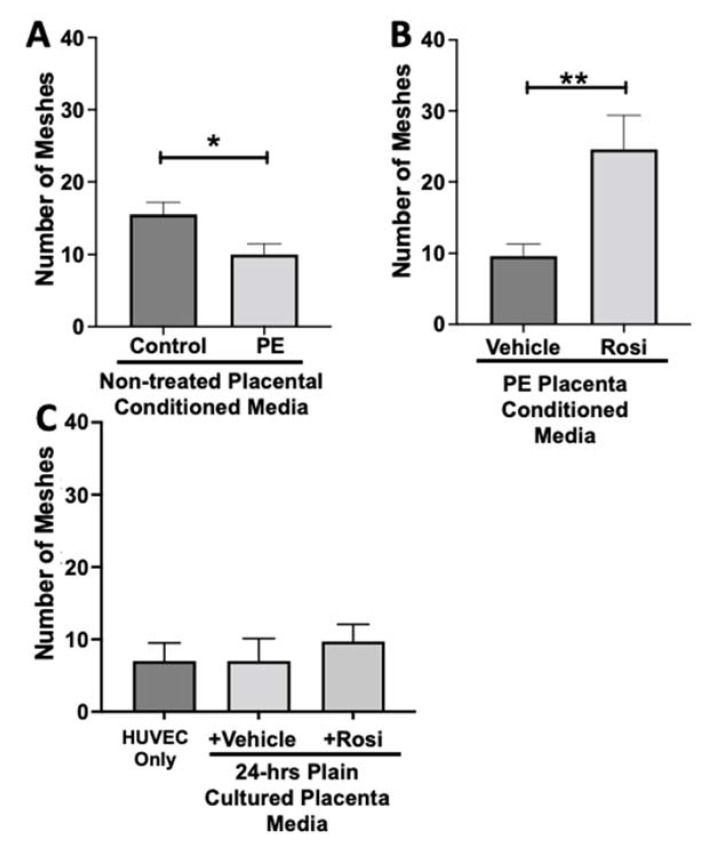
There is a reduction of the total number of meshes present in HUVECs from culture with conditioned media from preeclamptic placentas, but this was significantly increased after culture with Rosiglitazone-treated preeclamptic placentas. HUVECs were cultured with conditioned media on matrigel, and the number of meshes was calculated by the Image J Angiogenesis Analyzer tool (30). Conditioned media from preeclamptic (PE) placentas show significantly reduced the number of meshes as compared to healthy control placentas (**A**, n = 6). Conditioned media from Rosiglitazone (Rosi)-treated PE placentas led to a significant increase in the number of meshes present in the HUVECs compared to the conditioned media from the vehicle-treated PE placentas (**B**, n = 6). Rosi and vehicle were cultured in placental media without any tissues for 24 h along with HUVECs cultured with standard full-serum media all served as controls for this experiment. There were not any significant changes in the number of meshes among the HUVEC Only control, Rosi, and vehicle conditioned media controls (**C**, n = 6). (Statistical analysis was performed by student’s *t*-test to determine significant differences between groups, * *p* < 0.05, ** *p* < 0.01, bar plots and data reported are reported as numerical values ± SEM).

The number of meshes appear to be significantly reduced when HUVECs were cultured with conditioned media from PE placentas as compared to the conditioned media from control placentas (10 ± 1.5 vs. 15.5 ± 1.6, n = 6, *p* = 0.0165, Figure 13A). Conditioned media from Rosiglitazone-treated PE placentas led to a remarkable increase in the number of meshes present in the HUVECs, as compared to the conditioned media from the vehicle-treated PE placentas (24.5 ± 4 vs. 9.6 ± 1.7, n = 6, *p* = 0.0032, Figure 13B). There were no significant changes in the number of meshes present in the among the HUVEC Only control and the vehicle and Rosiglitazone conditioned media controls (7 ± 2.5, 7 ± 3, 9.7 ± 2.3, n = 6, *p* > 0.05, Figure 13C). 

**Figure 14:**
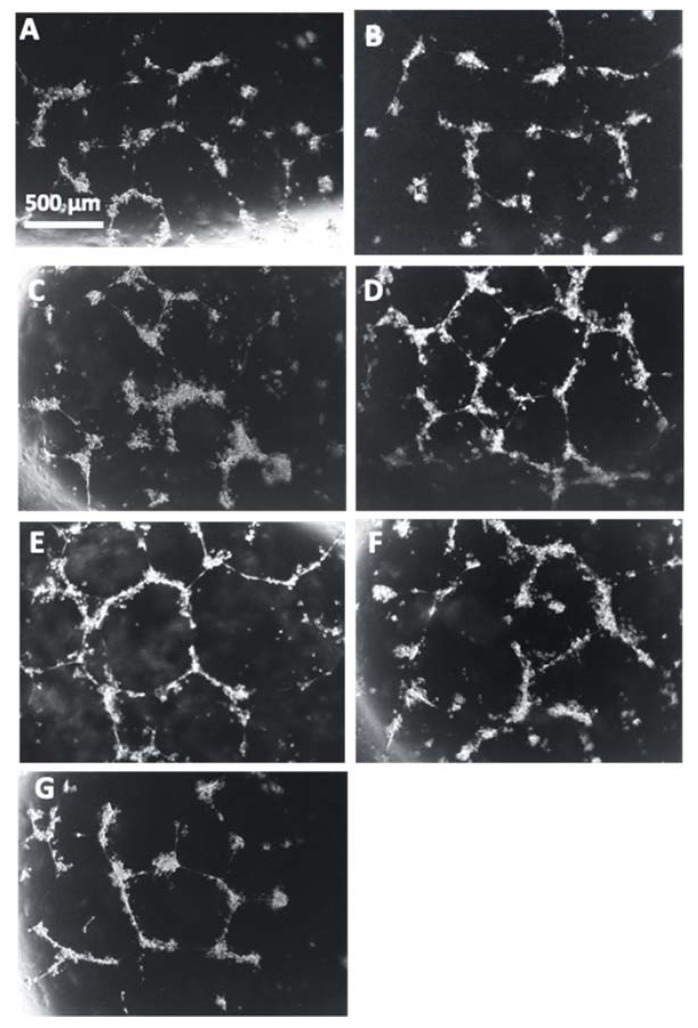
Representative images of HUVEC tube formation assays. Conditioned media from non-treated control (**A**) and preeclamptic (PE) (**B**) placentas, vehicle-treated PE placentas (**C**), Rosiglitazone (Rosi)-treated PE placentas (**D**), HUVEC Only control (**E**) and Rosi (**F**) and vehicle conditioned media controls (**G**) were cultured with HUVECs on matrigel and images were captured after 18 h of culture. Images were captured using a 4× magnification and then uploaded to the ImageJ Angiogenesis Analyzer to measure various parameters of tube formation to indicate which conditions permit the greatest angiogenic potential of the HUVECs. Scale bar represents 500 μm.

Representative images of the HUVECs with each treatment correlate with the reduction of angiogenic potential that was observed in the cells incubated with the conditioned media from PE placentas in comparison to the HUVECs cultured with conditioned media from control placentas (Figure 14A,B). There is a visible increase of tube formation observed in the HUVECs that were cultured with conditioned media from Rosiglitazone-treated PE placentas as compared to HUVEC culture with conditioned media from vehicle-treated PE placentas (Figure 14C,D). There appears to be no visible differences in tube formation in the HUVEC Only control or in the HUVECs cultured with either the vehicle-conditioned media control or Rosiglitazone-conditioned media control (Figure 14E–G). 

## 4. Discussion

Endothelial dysfunction is a major hallmark of PE which can cause severe symptoms in the mother that pose long-term risk of cardiovascular disease. In early onset PE and severe PE, the hypoxic and ischemic nature of the placenta is hypothesized to be a major contribution to the aberrant secretion of angiogenic and growth factor proteins that result in endothelial dysfunction [12,38,39,40]. There has been considerable evidence to show that PPARγ not only improves trophoblast function in ischemic placentas [22,23,41,42,43,44], but it can also influence the secretion of proteins that are important for maintaining an angiogenic balance, such as sFLT1 [25]. 

We questioned whether altered PPARγ activity and expression also drives aberrant placental protein secretion that leads to an overall anti-angiogenic state in the surrounding endothelium. We hypothesized through restoring placental expression of PPARγ, this could rescue the imbalance of angiogenic/growth factor proteins secreted from the PE placenta to subsequently lead to improved angiogenesis in the endothelium. To test this, we measured multiple angiogenic/growth factor proteins from healthy control and PE placentas as well as PE placentas that were treated with Rosiglitazone or vehicle to better understand the secretory profile in control versus PE placentas and to learn how these factors are influenced from placental activation of PPARγ. We further cultured human umbilical vein endothelial cells with conditioned media from these pregnancies to understand the overall ‘angiogenic potential’ of the secreted factors.

Angiopoietin 2 (Ang-2) is an important angiogenic protein that belongs to the angiopoietin/Tie2 pathway and is necessary for endothelial cell (EC) survival, maturation and morphogenesis [45]. Ang-2 is most known for its anti-angiogenic roles through serving as an antagonist to Angiopoeitin-1 (Ang-1) by competing for interaction with the EC surface receptor, Tie2 [45,46,47]. Ang-1 is very important for the reorganization of ECs, promoting structural integrity of the blood vessels and preventing EC leakage and migration of leukocytes to surrounding tissues by inhibiting EC activation [45], whereas blocking these effects through Ang-2 can largely contribute to vascular disease. In pregnancy, Ang-2 is mainly produced by the placenta and regulates EC survival, angiogenic sprouting and vascular regression [45,46]. Our Luminex data shows there is not much change in Ang-2 secretion between control and PE placentas. These results do not clarify prior reports in the literature. Some studies report that maternal plasma Ang-2 levels are increased in healthy pregnancies, as compared to non-pregnancy and post-partum women [48]. However, there have been conflicting evidence reporting Ang-2 levels in PE. Some studies show that maternal blood plasma Ang-2 levels are decreased in PE [48] while others describe an increase of Ang-2 placental mRNA expression and higher maternal Ang-2 plasma levels in PE compared to healthy pregnancies [49]. Furthermore, additional research suggests that measuring the Ang-1/Ang-2 ratio could be a method for predicting sePE onset, as the Ang-1/Ang-2 ratio has been shown to decrease during 25–28 weeks of gestation in women who later developed sePE [50]. Prior to this study, there has been little investigation on the role for PPARγ in regulating the expression of Ang-2. Our study is the first to report that placental activation of PPARγ by Rosiglitazone leads to a significant reduction of Ang-2 protein secretion. While this may seem like an exciting result, some studies have shown that intrauterine growth restricted (IUGR) pregnancies are associated with reduced Ang-2 levels, which could potentially interfere with placental angiogenesis [46]. More research is needed to understand the effects of PPARγ on Ang-2 and to determine healthy versus pathologic expression of Ang-2 in pregnancy.

Endothelial vasodilation is a crucial aspect to maintaining a steady and low-pressure flow of maternal blood to the implantation site that, when disrupted, can contribute to placental ischemia [51]. Soluble Endoglin (sEng) significantly impairs vasodilation and our Luminex data is in accordance with the literature in showing that there is a significant upregulation of sEng from the preeclamptic placentas in comparison to the healthy control placentas [52,53]. We excitedly found that sEng secretion is dampened in the PE placenta through PPARγ activation. This could be very beneficial in PE due to the significant contributions of sEng on endothelial dysfunction in PE. 

Endothelin-1 (ET-1) is another potent vasoconstrictive molecule that is known to be elevated in PE and contribute to endothelial dysfunction in multiple cardiovascular diseases [54,55,56]. We surprisingly did not observe a significant difference in ET-1 secretion from control placentas compared to preeclamptic placentas. One explanation for a lack of increased secretion in PE could be due to the placenta not serving as the primary source of ET-1 during pregnancy. Due to Rosiglitazone-treated PE placentas not showing any change in ET-1 levels, this result could suggest that placental PPARγ activation does not affect ET-1 secretion. There is little research that has investigated PPARγ regulation of ET-1 in the human placenta. However, there are reports in the literature that suggest in endothelial cells, PPARγ can regulate upstream pathways of ET-1 such as though increasing the expression of ET-1-inhibiting miRNAs, which result in reduced ET-1 expression [57]. It has also been shown that treatment with PPARγ agonists led to inactivation of the Activating Protein-1 (AP1) pathway which then led to transcriptional inactivation of ET-1 and subsequent reduction of ET-1 secretion [58]. It is possible that PPARγ activation could affect ET-1 at the transcript levels, but this may not be great enough to measure changes in protein secretion. 

Placental growth factor (PlGF) is another major factor in the regulation of angiogenesis [59]. While our data is not statically significant, it does follow a pattern of reduced PlGF secretion from the PE placenta and is increased in PE placentas treated with Rosiglitazone. Our data supports findings in the literature that discuss PlGF being downregulated in PE [60]. There is considerable evidence to suggest PPARγ may be able to indirectly regulate PlGF, due to the role for PPARγ to regulate the expression of GCM1 which acts directly upstream of PlGF to induce transcription [61,62,63]. 

Fibroblast growth factor-2 (FGF-2) has significant roles in regulating angiogenesis both in the endothelium and in the placenta [64]. FGF-2 has a direct role in the production of NO, which is very important since NO is one of the main vasodilatory agents in the placenta that contributes to regulation of trophoblast invasion, uterine vascular remodeling, and placental perfusion [65]. Our data shows that FGF-2 is decreased in PE. Although this result was not statistically significant, it does correlate with reports in the literature stating that women with sPE are shown to have significantly reduced blood serum levels of FGF-2 compared to women of healthy pregnancies [66]. Given the many roles of FGF-2 in the placenta and endothelium, increasing its production in PE would likely have many beneficial effects. Previous studies have shown that other PPARγ agonists in the TZD drug-family are shown to increase FGF-2 secretion from osteoblasts [67], however our study still brings novel information from the upregulation of FGF-2 secretion in the placenta from Rosiglitazone treatment. 

Placental function is also mediated through epidermal growth factor (EGF) and heparin-binding epidermal growth factor (HB-EGF) which both act on EGF receptors on the trophoblast and in the decidua [68,69,70,71]. There were no significant changes in EGF secretion between PE and control placentas, which is contradictory to reports in the literature that state EGF is decreased in PE [72]. It is however possible that the placenta may not be the main source of EGF production which could explain our results. We did observe significant reductions of HB-EGF from the PE placenta which is in accordance with reports in the literature [68,70]. The reduction of HB-EGF in term PE placentas may contribute to the enhanced trophoblast apoptosis. Fortunately, our data shows that Rosiglitazone treatment significantly increase HB-EGF secretion from the placenta, which could help to promote trophoblast cell survival in the PE placenta. This is an exciting finding can also confirm data reported by Kushwaha et al., who previously found that Rosiglitazone can increase HB-EGF in astrocytes [73]. 

Follistatin (FST) has important roles throughout the menstrual cycle such as through preventing hormone release to prevent follicular development [74,75,76]. Throughout pregnancy, FST levels generally increase then decrease towards normal levels within a few days following parturition [76]. We observed a significant reduction of FST from the PE placenta. While there is little data to show FST levels in during PE, it is known that FST levels are reduced during miscarriages [77,78] and implantation failure in IVF [79]. We observed a significant increase in FST levels when the PE placenta was treated with Rosiglitazone. This was a surprising finding, due to reports stating that PPARγ activation downregulates FST in intestinal epithelial cells [80]. The potential for PPARγ-upregulation of follistatin should be further investigated not only in the PE placenta, but also in the first trimester based on the known importance of FST functions in early pregnancy. 

Leptin is an important metabolic molecule that is known to be increased throughout healthy pregnancy [81,82]. We did not observe significant changes in leptin expression between PE and control placentas. Reports of leptin measurements in PE pregnancies do not all follow one pattern. Some studies mention there is an increase of leptin in women with late on-set PE, while others mention that early on-set PE have greater leptin expression even compared to late on-set PE [83]. There were no significant changes for leptin secretion based on Rosiglitazone treatment, which is surprising because other studies have reported that PPARγ and leptin can both enact on each other to reduce each other’s expressions in chondrocytes [84].

Collectively, the Luminex assay results supports the notion that the PE placenta exhibits greater secretions of anti-angiogenic proteins compared to controls, evidenced by the increase in sEng secretion and the decrease in PlGF, FGF-2 and HB-EGF secretion. We found that placental activation of PPARγ has an overall beneficial effect on the angiogenic profile through the reduction of Ang-2 and sEng and the upregulation of PlGF, FGF-2, HB-EGF and FST. To greater understand the impact of the angiogenic secretory profile influenced by placental activation of PPARγ, we used the conditioned medium from these placentas in culture with HUVECs. 

Endothelial cells undergo angiogenesis to form new blood vessels from existing blood vessels which occur in multiple conditions, such as hypoxia and during wound healing [30]. Angiogenesis must be initiated by a stimulus, often VEGF-A, which causes endothelial activation, degradation of the basement membrane, proliferation, and migration of the cells to form into tube-like structures. Using the Angiogenesis Analyzer tool [30], the phase contrast images captured from the tube formation assay are transformed and characteristic points and elements from the images are extracted and quantified. The nodes from the HUVEC structure represent the location of two branches and junctions are determined as a node with three or more branches that are intersecting. The total branching length is quantified as the sum of all the branch lengths per image. The ‘mesh’ is used to describe the capillary-like HUVEC structure and is measured by the areas enclosed by the branches. These measurements are often used in the literature to describe the potential for the HUVECs to undergo tube formation [85], which we refer to as ‘angiogenic potential’. 

Our data shows a very clear pattern of reduced number of nodes, junctions, total branching length and meshes in the HUVECs which were cultured with conditioned media from preeclamptic placentas. These data are not a surprise and confirm the claims presented in the literature that state the preeclamptic placenta causes an overall anti-angiogenic state. Remarkably, we saw that the Rosiglitazone-treated PE placentas cause overall greater number of nodes, junctions, total branching length and meshes, in comparison to the vehicle-treated PE placentas. This finding further validated our Luminex findings which had suggested an increase towards pro-angiogenic state of the PE placenta when treated with Rosiglitazone. 

We used several controls to ensure the changes in the HUVECs were due to indirect effects from the human placenta and not from residual drugs retained in the conditioned media (please see Table 2). To determine if there could be an effect on the HUVECs from any potential Rosiglitazone or vehicle retained in the placental conditioned media, we generated drug controls that consisted of human placenta culture media (without tissue) that was cultured overnight with Rosiglitazone or vehicle and then was applied to the HUVECs. We did not observe significant effects on the HUVECs from these conditioned media drug controls, thus we are confident the effects we did observe were due to proteins secreted from the human placenta. Moreover, HUVECs were serum-starved for 6 h prior to culture with the placental conditioned media, which was intended to halt HUVEC cell growth and proliferation, which further supports our findings that there was a placental-dependent effect on the HUVECs. In the ‘HUVEC Only’ samples, we applied the standard HUVEC culture medium that contained 10 % FBS and the required growth supplements, and we naturally observed that these cells formed capillary-tube-like structures. 

To our knowledge, we report novel findings of PPARγ actions in the placenta which have dramatic indirect effects on the endothelium. Future studies should follow up on the ability for PPARγ to modulate secreted proteins from the placenta such as Ang-2, sEng, PlGF, FGF-2, HB-EGF and FST. While VEGF-A was in our panel of markers to investigate, we did not obtain data that was within the standard curve from the Luminex assay. Due to the significant impacts of VEGF-A on the endothelium and initiation of angiogenesis, it would be helpful to know if placental activation of PPARγ effects VEGF-A secretion and thus should be investigated in a future follow-up study. Moreover, more detailed studies investigating the overall impact on the endothelial cells are warranted. For example, performing RNA-sequencing of the HUVEC cells would provide significant detail on the molecular mechanisms that are altered in these cells to permit the increased angiogenic potential after exposure to the human placental conditioned media. Lastly, it is important to note that the placenta secretes many proteins and that the combination of a variety of proteins determines the net effect on the endothelial cells. Therefore, we are unable to determine at this point if one or multiple proteins are responsible for the described phenotype. Nevertheless, the overall finding that the preeclamptic placenta can be treated ex vivo to impact the surrounding angiogenic potential deserves further attention due to the clinical significance of dampening endothelial dysfunction in the mother. 

## Figures and Tables

**Table 1:** Clinical Information.

Diagnosis	Gestation Age	Mode of Delivery	Maternal Age
PE	37wks	C-section	32
PE	39wks	C-section	33
PE	37wks	Vaginal	32
PE	38wks	C-section	22
PE	37wks	Vaginal	26
PE	37wks	Vaginal	33
PE	35wks	Vaginal	29
PE	37wks	Vaginal	37
PE	37wks	C-section	29
PE	31wks	C-section	19
sePE	39wks	C-section	37
sePE	37wks	Vaginal	20
sePE	39wks	Vaginal	20
sePE	36wks	Vaginal	19
Healthy control	40wks	Vaginal	33
Healthy control	39wks	Vaginal	25
Healthy control	39wks	Vaginal	28
Healthy control	39wks	Vaginal	28
Healthy control	36wks	C-section	29
Healthy control	36wks	Vaginal	28
Healthy control	35wks	Vaginal	33
Healthy control	36wks	C-section	34
Healthy control	37wks	Vaginal	36
Healthy control	37wks	C-section	26

**Table 2:** Experimental Conditions and Controls for HUVEC Tube Formation Assay.

Experimental Treatment	Culture Conditions
HUVEC Only	Standard culture medium
Vehicle Control	Placental culture medium (without human tissue) supplemented with DMSO and cultured for 24 h
Rosiglitazone Control	Placental culture medium (without human tissue) supplemented with Rosiglitazone and cultured for 24 h
Conditioned medium from non-treated preeclamptic placentas	Preeclamptic tissue cultured in placental culture medium for 24 h
Conditioned medium from preeclamptic placentas treated with vehicle	Preeclamptic tissue cultured in placental culture medium for 24 h with DMSO
Conditioned medium from preeclamptic placentas treated with Rosiglitazone	Preeclamptic tissue cultured in placental culture medium for 24 h with Rosiglitazone
Conditioned medium from non-treated healthy control placentas	Healthy placentas cultured in placental culture medium from 24 h

## Data Availability

Not applicable.
